# Monitoring and Characteristics of Major Mastitis Pathogens from Bulk Tank Milk in Korea

**DOI:** 10.3390/ani10091562

**Published:** 2020-09-02

**Authors:** Mun-Jo Yun, Sunghyun Yoon, Young Ju Lee

**Affiliations:** 1 College of Veterinary Medicine & Zoonoses Research Institute, Kyungpook National University, Daegu 41566, Korea; yun200297@naver.com (M.-J.Y.); sungyoon@knu.ac.kr (S.Y.); 2 Gyeongsangbuk-do Provincial Government Office, Andong 36759, Korea

**Keywords:** bulk tank milk, mastitis pathogen, antimicrobial resistance, somatic cell count

## Abstract

**Simple Summary:**

The monitoring of milk quality and the presence of major mastitis pathogens is an important part of milk quality assurance program. Bulk tank milk has been used in many countries to identify multiple milk quality problems and mastitis pathogens that might exist in a dairy herd. This study aimed to compare the presence of mastitis pathogens and the antimicrobial resistance of the isolates from bulk tank milk by dairy companies. The results showed that the prevalence of mastitis pathogens and the antimicrobial resistance of the isolates were significantly different among factories, and support the development of strong monitoring and prevention programs in dairy operations.

**Abstract:**

In many countries, bulk tank milk (BTM) has been used for examining milk and analyzed as an important part of milk quality assurance programs. The objectives of this study were to investigate milk quality and the presence of major mastitis pathogens in BTM, and to compare the characteristics of BTM by dairy factory or company. A total of 1588 batches of BTM samples were collected from 396 dairy farms of seven dairy factories owned by four companies in Korea. The means of individual bacterial counts (IBC) and somatic cell count (SCC) were 3.7 × 10^4^ cells/mL and 1.1 × 10^5^ cells/mL, respectively, and no significant differences among dairy factories were observed. The most common pathogen was *Staphylococcus* spp. (60.1%), followed by *E. faecalis* (53.8%), *E. coli* (37.6%) and *Streptococcus* spp. (22.5%). *Enterococcus* spp. showed the highest resistance to tetracyclines (51.1% to 73.9%) and macrolides (46.5%). *S. aureus* and coagulase-negative staphylococci (CNS) showed the highest resistance to penicillin (28.4% and 40.2%, respectively), and three (3.2%) *S. aureus* and seven (3.3%) CNS were also methicillin-resistant. These data show the diverse prevalence and characteristics of major mastitis pathogens among factories, and support the development of strong monitoring and prevention programs of mastitis pathogens by commercial dairy operations.

## 1. Introduction

Bulk tank milk (BTM) has been used for identifying multiple milk quality problems and mastitis pathogens that might exist in a dairy herd. It is analyzed as an important part of milk quality assurance programs in many countries [1,2,3,4]. Microbiological screening of all cows in a herd can be laborious and expensive, so performing tests using BTM is convenient and economical [5]. The isolation of mastitis pathogens from BTM indicates that contamination or intramammary infection is present in one or more cows on the farm [6], so monitoring BTM samples is useful in controlling mastitis on dairy farms because early detection of the causative pathogens is the most effective way to prevent disease and guide treatment [7].

In most countries, the bacterial quality of BTM is monitored by individual bacterial counts (IBC) and somatic cell count (SCC) [8,9]. In particular, the majority of somatic cells are leukocytes, which increase in milk, usually as an immune response to mastitis. A threshold SCC of 2 × 10^5^ cells/mL would determine the financial penalty of milk, and an SCC of more than 5 × 10^5^ cells/mL makes milk deemed unfit for human consumption in Korea [10]. Not only the contagious pathogens, such as *S. aureus* and *Streptococcus* spp., which can be transferred from cow to cow, but also environmental pathogens such as *E. coli*, *Enterococcus* spp. and coagulase-negative staphylococci (CNS), which are considered opportunistic pathogens responsible for subclinical mastitis, can be spread to other cows through a contaminated environment in the barn and the milking process [11].

Antimicrobial therapy is common in Korea, which is one of the key components for the control of infectious mastitis, so the emergence of the antimicrobial resistance in major mastitis pathogens such as methicillin-resistant *S. aureus* (MRSA) has been reported [12], and is a public health concern [13]. The dairy farm environment has also been reported as a reservoir for the distribution of antimicrobial resistance [14]. The purpose of this study was to investigate the milk quality and the presence of contagious and environmental mastitis pathogens, such as *E. coli*, *Enterococcus* spp., *S. aureus*, CNS and *Streptococcus* spp. in BTM, which consists of raw milk from cows of dairy farms where mastitis has not been detected, and to compare the characteristics of BTM by dairy factory or company. To our knowledge, there are no reports on monitoring and comparing the quality of BTM among dairy factories in Korea.

## 2. Materials and Methods

### 2.1. Collection of Samples

A total of 1588 batches of BTM were collected from 396 dairy farms of seven dairy factories operated by four companies in Korea. Milk samples, 50 mL each, were aseptically collected two times in the summer and winter season during the study period (July–December 2019) and sent to the laboratory under 4 °C conditions. All samples were then individually tested for IBC, SCC and identification of bacterial isolates within 24 h.

### 2.2. IBC and SCC

All batches of BTM were analyzed for IBC and SCC using BactoScan FC (Foss Electric, Hillerød, Denmark) and Milkoscan CombiFoss 6000 (Foss Electric, Oceanside, CA, USA), respectively.

### 2.3. Bacterial Identification

The isolation and identification of mastitis pathogens were performed following the standard microbiological protocols published by the Ministry of Food and Drug Safety (2018) [15]. Among the many mastitis pathogens, *E. coli*, *Enterococcus* spp., *Staphylococcus* spp. and *Streptococcus* spp. were investigated in this study. Briefly, one mL of each milk sample was aerobically cultured in 9 mL of mEC (Merck, Darmstadt, Germany), tryptic soy broth with 6% NaCl (BD Biosciences, Sparks, MD, USA) and Todd Hewitt broth (BD Biosciences) for *E. coli*, *Staphylococcus* spp. and *Streptococcus* spp., respectively. After incubation at 37 °C for 24 h, each medium was streaked onto MacConeky agar (BD Biosciences), Baird-Parker agar (BD Biosciences) and 5% sheep blood agar (KOMED, Seoul, Korea), respectively. For isolation of *Enterococcus* spp., one mL of the milk sample was cultured in 9 mL of buffered peptone water (BPW; BD Biosciences). Then, pre-enriched BPW was mixed with Enterococcosel broth (BD Biosciences) at a 1:10 ratio. After incubation at 37 °C for 18–24 h, each medium was streaked onto Enterococcosel agar (BD Biosciences). Suspected colonies were transferred to each selective medium for bacterial identification. Confirmation of *E. coli*, *E. faecalis*, *E. faecium*, *S. aureus* and *Streptococcus* spp. was performed using PCR with specific primers (Table 1). Classification of CNS spp. and *Streptococcus* spp. was performed by MALDI-TOF mass spectrometry (Biomerieux, Marcy-l’Étoile, France) based on the protein expression profiles using VITEK MS system (Biomerieux). If two isolates of the same origin showed the same antimicrobial susceptibility patterns, only one isolate was randomly chosen and included in this study.

### 2.4. Antimicrobial Susceptibility Testing

Based on the Clinical and Laboratory Standards Institute guidelines (CLSI, 2019) [20], all pathogens were investigated for antimicrobial resistance using the disc diffusion test with the following discs (BD Biosciences): amikacin (A, 30 µg), ampicillin (AM, 10 µg), amoxicillin-clavulanate (AMC, 20 µg), chloramphenicol (C, 30 µg), ceftazidime (CAZ, 30 µg), clindamycin (CC, 2 µg), cefadroxil (CDX, 30 µg), cephalothin (CF, 30 µg), ciprofloxacin (CIP, 5 µg), colistin (CL, 10 µg), cefotaxime (CTX, 30 µg), cefuroxime (CXM, 30 µg), cefazoline (CZ, 30 µg), doxycycline (DOX, 30 µg), erythromycin (E, 15 µg), nitrofurantoin (F/M, 300 µg), cefepime (FEP, 30 µg), cefoxitin (FOX, 30 µg), gentamicin (G, 10 µg), imipenem (IPM, 10 µg), kanamycin (K, 30 µg), levofloxacin (LVX, 5 µg), nalidixic acid (Na, 30 µg), norfloxacin (NOR, 10 µg), ofloxacin (OFX, 5 µg), oxacillin (OX, 1 µg), penicillin (P, 10 units), rifampin (RA, 5 µg), trimethoprim/sulfamethoxazole (SXT, 1.25 µg), tetracycline (TE, 30µg), teicoplanin (TEC, 30µg) and vancomycin (VA, 30 µg). Methicillin resistance in *S. aureus* and CNS isolates was determined by the results of the cefoxitin disk diffusion test as recommended by CLSI (2019) [20] and Pourmand et al. (2014) [21]. *E. coli* ATCC 25922, *E. faecalis* ATCC 29,212 and *S. aureus* ATCC 29,213 were used as the quality controls. Multidrug resistance (MDR) was defined as acquired resistance to at least one agent in three or more antimicrobial classes [22].

### 2.5. Statistical Analysis

Statistical analyses were performed using SPSS 25 (IBM Corp., Armonk, NY, USA). The analysis of the differences of the means of IBC and SCC samples collected from each dairy factory were carried out by a logarithmic transformation of values with log base 10 as reported by Lopes Júnior (2012) and conducted by one-way ANOVAs [8]. If a significant difference (*p* < 0.05) in the prevalence of pathogens among factories was confirmed by the Chi-square test, post-hoc analyses using the Bonferroni correction were performed for multiple tests in subsequent analyses of the seven dairy factories, as previously reported [23].

## 3. Results

### 3.1. Comparison of IBC and SCC

Comparisons of IBC and SCC in BTM from 396 farms of seven dairy factories are shown in Table 2. Means of log_10_IBC and log_10_SCC were 4.38 ± 0.53–4.57 ± 0.41 and 4.94 ± 0.39–5.10 ± 0.46, respectively. The means of IBC from four of the seven factories were greater than 3.0 × 10^4^ cells/mL.

### 3.2. Distribution of Major Mastitis Pathogens

The distributions of pathogens in BTM are shown in Table 3. Although the most common pathogens were *Staphylococcus* spp. (238 farms, 60.1%), the prevalence of CNS (164 farms, 41.4%) was higher than that of *S. aureus* (95 farms, 24.0%). The prevalence of *E. faecalis, E. coli* and *Streptococcus* spp. was 53.8%, 37.6% and 22.5%, respectively. In the distribution of pathogens among dairy factories, *E. coli* and *S. aureus* were not detected from factories A-1 and A-2 and factory B-1. Moreover, the prevalence of *E. coli, Enterococcus* spp. and *S. aureus* was significantly different among factories by application of the Bonferroni correction. In particular, the prevalence of *E. coli* from factories D-2 and D-3 and *E. faecalis* from factory D-3 was significantly higher than in the other factories, but factory A-2 had a significantly higher prevalence of *S. aureus* than other factories had.

### 3.3. Antimicrobial Resistance

Antimicrobial resistance patterns of 918 pathogens isolated from BTM are shown in Table 4. While *E. coli* had the lowest resistance, from 0% to 13.1%, to all antimicrobials tested, *Enterococcus* spp. had the highest resistance to tetracyclines (51.1% to 73.9%) and macrolides (46.5%). *S. aureus* and CNS showed the highest resistance to penicillin (28.4% and 40.2%, respectively), and three (3.2%) *S. aureus* and seven (3.3%) CNS also showed methicillin resistance. *Streptococcus* spp. had the highest resistance to tetracycline (49.5%), followed by clindamycin (35.5%) and cefotaxime (24.7%).

Distribution of MDR pathogens is shown in Table 5. The highest prevalence of MDR was found in *Streptococcus* spp. (29.0%), followed by *Enterococcus* spp. (24.3%), CNS (16.4%) and *E. coli* (13.5%). The prevalence of MDR was lowest in *S. aureus* (2.1%). MDR to six antimicrobial classes was found in two *E. coli*, one CNS and one *S. aureus*, which were the isolates with an intermediate prevalence of MDR.

## 4. Discussion

The IBC and SCC, which are used as general indicators of milk safety, did not show significant differences among factories (*p* = 0.219 and *p* = 0.418, respectively), which can be influenced by cow health, environment, milking procedures and equipment sanitation [24]. In Korea, most of the milk products are produced by five large dairy companies, which account for 84% of the total sales of dairy products in Korea [25]. They control and operate in all phases of the dairy production system [26]. Additionally, most dairy farms consist of herds living in confined space, resulting in a higher chance of contamination by environmental pathogens on teats of cows, as reported by Goldberg et al. (1992) [27]. In this study, the means of IBC of BTM samples from seven factories originating from four dairy companies ranged from 2.4 × 10^4^ to 3.7 × 10^4^ cells/mL. Although hygiene management and cow health have been continuously prioritized and financial penalties have been applied when the level of IBC is over 3.0 × 10^4^ cells/mL, the means of IBC from four out of seven factories were still over 3.0 × 10^4^ cells/mL. IBC gives an estimate of the total number of viable aerobic bacteria present in raw milk, and can be controlled by the consistent application of proper milking practices, udder hygiene and mastitis prevention [28]. A high level of IBC suggests that bacteria are entering the milk from a variety of possible sources. The most frequent cause is poor cleaning and cooling techniques of milking systems and milk residues on equipment surfaces, which provide nutrients for growth and multiplication of bacteria that contaminate subsequent milking [2]. Thus, dairy farms should improve control practices to reduce bacterial infection in milking processes, as previously reported [29].

The means of SCC of seven factories showed a relatively low level (8.6 × 10^4^–1.2 × 10^5^ cells/mL) compared to the established standard for financial penalties (2.0 × 10^5^ cells/mL). Additionally, the SCC of BTM samples had a lower level than that of raw milk samples in 2007 (2.4 × 10^5^ cells/mL) in Korea [30]. Although the lower SCC in BTM than the raw milk samples may be attributed to the dilution effect of milk masking a high SCC of individual cows [31], the BTM tested in this study may have consisted of raw milk from cows of dairy farms without clinical mastitis.

Although there were no significant differences in IBC and SCC among factories, there were significant differences in the prevalence of major mastitis pathogens, such as *S. aureus,* which might be caused by a relatively low level of pathogens in BTM, as previously reported by Fenlon et al. (1995) and Koop et al. (2010) [32,33]. In this study, the prevalence of *E. faecalis* and *E. coli* was 53.8% and 37.6%, respectively, and the prevalence in factories of D company, in particular, was significantly higher, although *E. coli* were not detected at all in the factories of A company. *E. faecalis* and *E. coli* are considered to indicate fecal contamination, produced under poor hygienic conditions [29]. The results in factories, including company D, may reflect poor management of fecal contamination, the most common source of pathogens in BTM [29]. Moreover, the prevalence of *S. aureus* was also significantly different among factories, and the A-2 factory of A company had a significantly higher prevalence than other factories. Although the samples from factory A-2 also showed low SCC, continuous monitoring of SCC is required, because *S. aureus* can lead to recurrent infection and shed in a cyclical way from mammary glands, which can result in clinical mastitis [34].

In this study, the prevalence of CNS and *Streptococcus* spp. was 41.4% and 22.5%, respectively. The presence of these pathogens does not necessarily induce inflammation [35], but it should be also monitored and controlled, because they are opportunistic pathogens that have the potential to be hazardous [7], causing clinical signs in a susceptible host [36].

Although the samples tested in this study consisted of raw milk from cows without clinical mastitis, the highest prevalence of antimicrobial resistance was found in *Enterococcus* spp. (88.2%), followed by CNS (64.5%), *Streptococcus* spp. (60.2%), *E. coli* (40.0%) and *S. aureus* (31.6%). In particular, antimicrobial resistance to tetracycline was relatively widespread in *Enterococcus* spp. (73.9%) and *Streptococcus* spp. (49.5%), as previous studies in Korea have shown [37,38]. Additionally, the resistance to penicillin was also high in *S. aureus* (28.4%) and CNS (40.2%), consistent with previous studies [37,38,39]. The prevalence of resistance to penicillin and tetracycline may be associated with the heavy use of these antimicrobials in the food animals, because these antibiotics have been reported to be the most consumed antimicrobials in Korea [40].

Cefoxitin resistance, which is considered as methicillin resistance in the CLSI (2019) guidelines, was found in three *S. aureus* and seven CNS [21]. MRSA and methicillin-resistant CNS are problematic, not only in the dairy industry but also in public health generally, because they cause resistant *Staphylococcus* spp. infections in humans [21,41]. Notably, two vancomycin-resistant isolates were also found in this study, which is concerning because of the limited therapeutic choices beyond vancomycin for treating infections with these organisms [42]. Therefore, further investigations on the risks to personnel on dairy farms should be conducted.

In addition, *Streptococcus* spp. was highly resistant to clindamycin (35.5%) and this percentage was slightly higher than in reports from China (28.7%) and Argentina (25.5%) [13,43]. Resistance to chloramphenicol was also found in all mastitis pathogens in this study (2.1–26.1%), as other studies in Korea have reported [38,44]. However, clindamycin has not been approved for use in cattle, and chloramphenicol has been withdrawn from food animal use in Korea since 1992 [45]. Phenicol antimicrobials, such as chloramphenicol and florfenicol, and lincosamide antimicrobials, such as clindamycin, are potent inhibitors of bacterial protein biosynthesis [46]. Although chloramphenicol and clindamycin have not been used in veterinary medicine, florfenicol has been widely used for veterinary medicine in Korea [47]. Resistance to phenicols and lincosamides could be mediated by rRNA methyltransferase, which modifies RNA in the drug-binding site [46] and chloramphenicol, florfenicol and clindamycin showed partially overlapped drug binding sites. Therefore, the use of florfenicol can confer resistance to chloramphenicol and clindamycin [46,48].

The highest prevalence of MDR was seen in *Streptococcus* spp. (29.0%), followed by *Enterococcus* spp. (24.3%), CNS (16.4%), *E. coli* (13.5%) and *S. aureus* (2.1%). Among them, 10 isolates showed resistance to six antimicrobial classes. Although the use of antimicrobials in the dairy industry in Korea is strictly monitored and managed, the emergence of antimicrobial-resistant pathogens in milk is of concern, because antimicrobial-resistant genes and mobile genetic elements, such as transposons, may be disseminated nationwide and transferred horizontally through mastitis pathogens [44,49].

## 5. Conclusions

Although there were no significant differences among factories in regards of IBC and SCC, the prevalence of pathogens and the antimicrobial resistance of them showed significant differences among factories, which could be affected by the management programs of dairy factories and companies. To prevent the emergence of the mastitis pathogens and its antimicrobial resistant properties, strong monitoring and prevention programs of mastitis pathogens should be implemented among commercial dairy operations.

## Figures and Tables

**Table 1:** PCR primers used in this study.

Target Microorganism	Target Gene	Primer	Sequence (5′–3′)	Amplicon Size (bp)	Annealing Temperature (°C)	References
*E. coli*	*malB*	malBF	TCGCCACACGCTGACGCTGACCA	585	55	[16]
		malBR	TTACATGACCTCGGTTTAGTTCACAGA			
*E. faecalis*	*ddl1*	ddl1F	TGTTGTATGGCGGCAGAAGT	941	54	[17]
		ddl1R	TCAGGTGTTTGTGCCCAAGT			
*E. faecium*	*ddl2*	ddl2F	ATGGGACCCAAGTGGACAGA	550	54	[17]
		ddl2R	ATTTCGCGCGCTTCAATTCC			
*S. aureus*	*nuc*	NucF	GCGATTGATGGTGATACGGTT	279	55	[18]
		NucR	AGCCAAGCCTTGACGAACTAAAGC			
*Streptococcus*	16S-23S rRNA	SU-F2	AGCCGCCTAAGGTGGGAT	220–230	60	[19]
		SU-R	ATGGAGCCTAGCGGGATC			

**Table 2:** Distribution of individual bacterial count (IBC) and somatic cell count (SCC) in bulk tank milk samples from dairy factories.

Company	Dairy Factory	Number of Bulk Tank Milk Samples ^a^	Log_10_IBC			Geometric Mean of IBC (cells/mL)	log_10_SCC			Geometric Mean of SCC (cells/mL)
			Mean ^b^	SD ^c^	CI ^d^		Mean ^e^	SD ^c^	CI ^d^	
A	A-1	200	4.38	0.53	4.23–4.53	24,039	5.02	0.48	4.88–5.16	105,024
	A-2	224	4.48	0.66	4.30–4.65	30,135	5.06	0.64	4.89–5.23	115,320
B	B-1	480	4.57	0.41	4.49–4.64	36,939	5.10	0.46	5.01–5.18	124,517
C	C-1	188	4.51	0.37	4.40–4.62	32,598	5.01	0.40	4.89–5.13	102,245
D	D-1	232	4.43	0.37	4.33–4.53	26,955	4.94	0.39	4.83–5.04	86,570
	D-2	132	4.40	0.44	4.25–4.56	25,405	4.97	0.43	4.82–5.12	92,614
	D-3	128	4.43	0.53	4.24–4.62	26,651	4.95	0.48	4.78–5.13	89,975
Total		1588	4.48	0.48	4.43–4.53	30,197	5.03	0.48	4.98–5.07	107,261

^a^ The bulk tank milk samples were collected two times, summer and winter (July to December 2019) from 396 dairy farms of seven factories of four dairy companies. ^b^ Means of log_10_IBC were not significantly different among factories (*p* = 0.219); ^c^ SD, standard deviation; ^d^ CI, confidence interval (95%); ^e^ Means of log_10_SCC were not significantly different among factories (*p* = 0.418).

**Table 3:** Species and distribution of pathogens isolated from 396 dairy farms among seven dairy factories.

Genus.	Species	No. of Pathogens Isolated (%) among Farms ^a^							Total (%) (*n* = 396)
		A-1 (*n* = 50)	A-2 (*n* = 56)	B-1 (*n* = 120)	C-1 (*n* = 47)	D-1 (*n* = 58)	D-2 (*n* = 33)	D-3 (*n* = 32)	
*Escherichia coli*		0 (0.0) *	0 (0.0) *	37 (30.8)	28 (59.6) *	29 (50.0)	26 (78.8) *	29 (90.6) *	149 (37.6)
*Enterococcus* spp.		20 (40.0)	19 (33.9) *	69 (57.5)	30 (63.8)	38 (65.5)	23 (69.7)	32 (100.0) *	231 (58.3)
	*Enterococcus faecalis*	18 (36.0)	19 (33.9) *	64 (53.3)	25 (53.2)	35 (60.3)	21 (63.6)	31 (96.9) *	213 (53.8)
	*Enterococcus faecium*	2 (4.0)	2 (3.6)	6 (5.0)	11 (23.4) *	6 (10.3)	2 (6.1)	1 (3.1)	30 (7.6)
*Staphylococcus* spp.		48 (96.0) *	52 (92.9) *	40 (33.3) *	24 (51.1)	48 (82.8) *	18 (54.5)	8 (25.0) *	238 (60.1)
	*Staphylococcus aureus*	19 (38.0)	52 (92.9) *	0 (0.0) *	3 (6.4) *	5 (8.6) *	13 (39.4)	3 (9.4)	95 (24.0)
Coagulase-negative staphylococci		37 (74.0) *	8 (14.3) *	40 (33.3)	22 (46.8)	44 (75.9) *	8 (24.2)	5 (15.6) *	164 (41.4)
	*Staphylococcus chromogenes*	35 (70.0) *	6 (10.7)	3 (2.5) *	2 (4.3)	12 (20.7)	4 (12.1)	1 (3.1)	63 (15.9)
	*Staphylococcus saprophyticus*	0 (0.0)	1 (1.8)	17 (14.2)	12 (25.5) *	14 (24.1) *	1 (3.0)	0 (0.0)	45 (11.4)
	*Staphylococcus xylosus*	1 (2.0)	1 (1.8)	6 (5.0)	3 (6.4)	6 (10.3)	0 (0.0)	0 (0.0)	17 (4.3)
	*Staphylococcus haemolyticus*	0 (0.0)	0 (0.0)	5 (4.2)	1 (2.1)	4 (6.9)	1 (3.0)	0 (0.0)	11 (2.8)
	*Staphylococcus simulans*	1 (2.0)	0 (0.0)	0 (0.0)	0 (0.0)	1 (1.7)	2 (6.1)	2 (6.3)	6 (1.5)
	*Staphylococcus sciuri*	0 (0.0)	0 (0.0)	2 (1.7)	2 (4.3)	1 (1.7)	0 (0.0)	0 (0.0)	5 (1.3)
	*Staphylococcus capitis*	0 (0.0)	0 (0.0)	2 (1.7)	0 (0.0)	1 (1.7)	0 (0.0)	0 (0.0)	3 (0.8)
	*Staphylococcus cohnii*	0 (0.0)	0 (0.0)	1 (0.8)	0 (0.0)	1 (1.7)	0 (0.0)	0 (0.0)	2 (0.5)
	*Staphylococcus epidermidis*	0 (0.0)	0 (0.0)	0 (0.0)	1 (2.1)	0 (0.0)	0 (0.0)	1 (3.1)	2 (0.5)
	*Staphylococcus equorum*	0 (0.0)	0 (0.0)	0 (0.0)	1 (2.1)	1 (1.7)	0 (0.0)	0 (0.0)	2 (0.5)
	*Staphylococcus gallinarum*	0 (0.0)	0 (0.0)	1 (0.8)	0 (0.0)	1 (1.7)	0 (0.0)	0 (0.0)	2 (0.5)
	*Staphylococcus succinus*	0 (0.0)	0 (0.0)	2 (1.7)	0 (0.0)	0 (0.0)	0 (0.0)	0 (0.0)	2 (0.5)
	*Staphylococcus hyicus*	0 (0.0)	0 (0.0)	1 (0.8)	0 (0.0)	0 (0.0)	0 (0.0)	1 (3.1)	2 (0.5)
	*Staphylococcus arlettae*	0 (0.0)	0 (0.0)	0 (0.0)	0 (0.0)	1 (1.7)	0 (0.0)	0 (0.0)	1 (0.3)
	*Staphylococcus lentus*	0 (0.0)	0 (0.0)	0 (0.0)	0 (0.0)	1 (1.7)	0 (0.0)	0 (0.0)	1 (0.3)
*Streptococcus* spp.		6 (12.0)	9 (16.1)	27 (22.5)	10 (21.3)	16 (27.6)	18 (54.5) *	3 (9.4)	89 (22.5)
	*Streptococcus bovis*	0 (0.0)	0 (0.0)	14 (11.7)	0 (0.0)	3 (5.2)	8 (24.2) *	0 (0.0)	25 (6.3)
	*Streptococcus uberis*	1 (2.0)	9 (16.1) *	3 (2.5)	3 (6.4)	1 (1.7)	0 (0.0)	2 (6.3)	19 (4.8)
	*Streptococcus oralis*	3 (6.0)	0 (0.0)	0 (0.0)	2 (4.3)	4 (6.9)	6 (18.2) *	1 (3.1)	16 (4.0)
	*Streptococcus infantarius*	0 (0.0)	0 (0.0)	4 (3.3)	0 (0.0)	0 (0.0)	3 (9.1) *	0 (0.0)	7 (1.8)
	*Streptococcus agalactiae*	1 (2.0)	0 (0.0)	0 (0.0)	0 (0.0)	0 (0.0)	0 (0.0)	0 (0.0)	1 (0.3)
	*Streptococcus intermedius*	0 (0.0)	0 (0.0)	1 (0.8)	0 (0.0)	0 (0.0)	0 (0.0)	0 (0.0)	1 (0.3)
	Unidentified	1 (2.0)	0 (0.0)	5 (4.2)	5 (10.6)	8 (13.8) *	1 (3.0)	0 (0.0)	20 (5.1)

^a^ Seven factories were operated by four dairy companies, and the same capital letter indicates factories operated by the same company. * The asterisk indicates that prevalence of the pathogen isolated from the factory was significantly different than that of other factories using the Bonferroni correction.

**Table 4:** Antimicrobial resistance in 918 pathogens isolated from bulk tank milk.

Antibiotic (Abbreviation)	No. of Resistant Isolates (%)				
	*E. coli* (*n* = 183)	*Enterococcus* spp. (*n* = 333)	*S. aureus* (*n* = 95)	CNS ^a^ (*n* = 214)	*Streptococcus* spp. (*n* = 93)
β-Lactams					
Penicillin (P)	N/A ^b^	5 (1.5)	27 (28.4)	86 (40.2)	N/A
Ampicillin (AM)	24 (13.1)	2 (0.6)	3 (3.2)	19 (8.9)	N/A
Amoxicillin/clavulanate (AMC)	5 (2.7)	N/A	2 (2.1)	12 (5.6)	N/A
Oxacillin (OX)	N/A	N/A	3 (3.2)	14 (6.5)	N/A
Methicillin (MET)	N/A	N/A	3 (3.2)	7 (3.3)	N/A
Cephems					
Cefazolin (CZ)	9 (4.9)	N/A	3 (3.2)	3 (1.4)	N/A
Cefadroxil (CDX)	5 (2.7)	N/A	N/A	N/A	N/A
Cephalothin (CF)	25 (14)	N/A	1 (1.1)	2 (0.9)	N/A
Cefuroxime (CXM)	3 (1.6)	N/A	1 (1.1)	3 (1.4)	N/A
Cefoxitin (FOX)	3 (1.6)	N/A	3 (3.2)	7 (3.3)	N/A
Ceftazidime (CAZ)	5 (2.7)	N/A	1 (1.1)	2 (0.9)	N/A
Cefotaxime (CTX)	3 (1.6)	N/A	1 (1.1)	3 (1.4)	23 (24.7)
Cefepime (FEP)	3 (1.6)	N/A	1 (1.1)	3 (1.4)	21 (22.6)
Glycopeptides					
Vancomycin (VA)	N/A	2 (0.6)	0 (0.0)	0 (0.0)	N/A
Teicoplanin (TEC)	N/A	N/A	0 (0.0)	1 (0.5)	N/A
Imipenem (IPM)	0 (0.0)	N/A	N/A	N/A	N/A
Aminoglycosides					
Gentamicin (G)	19 (10)	NA	1 (1.1)	3 (1.4)	N/A
Amikacin (A)	0 (0.0)	N/A	0 (0.0)	0 (0.0)	N/A
Kanamycin (K)	4 (2.2)	N/A	2 (2.1)	12 (5.6)	N/A
Macrolides					
Erythromycin (E)	N/A	155 (46.5)	2 (2.1)	13 (6.1)	17 (18.3)
Tetracyclines					
Tetracycline (TE)	26 (14.2)	246 (73.9)	2 (2.1)	65 (30.4)	46 (49.5)
Doxycycline (DOX)	9 (4.9)	170 (51.1)	0 (0.0)	12 (5.6)	N/A
Quinolones					
Nalidixic acid (Na)	2 (1.1)	N/A	N/A	N/A	N/A
Fluoroquinolones					
Ciprofloxacin (CIP)	2 (1.1)	13 (3.9)	1 (1.1)	1 (0.5)	N/A
Levofloxacin (LVX)	N/A	5 (1.5)	1 (1.1)	1 (0.5)	1 (1.1)
Norfloxacin (NOR)	N/A	5 (1.5)	2 (2.1)	1 (0.5)	N/A
Ofloxacin (OFX)	N/A	N/A	1 (1.1)	1 (0.5)	2 (2.2)
Nitrofurantoins					
Nitrofurantoin (F/M)	N/A	0 (0.0)	0 (0.0)	0 (0.0)	N/A
Lincosamides					
Clindamycin (CC)	N/A	N/A	1 (1.1)	8 (3.7)	33 (35.5)
Folate pathway inhibitors					
Trimethoprim/sulfamethoxazole (SXT)	7 (3.8)	N/A	1 (1.1)	1 (0.5)	0 (0.0)
Phenicols					
Chloramphenicol (C)	11 (6.0)	87 (26.1)	2 (2.1)	45 (21.0)	21 (22.6)
Ansamycins					
Rifampin (RA)	N/A	63 (18.9)	0 (0.0)	1 (0.5)	0 (0.0)
Polymyxins					
Colistin (CL)	16 (8.7)	N/A	N/A	N/A	N/A

^a^ CNS, coagulase-negative *Staphylococcus* spp. ^b^ N/A, not applicable based on CLSI (2019) guidelines.

**Table 5:** Distribution of multidrug resistance of 918 pathogens isolated from bulk tank milk.

No. of Resistant Antimicrobial Classes	No. of Isolates (%)				
	*E. coli* (*n* = 183)	*Enterococcus* spp. (*n* = 333)	*S. aureus* (*n* = 95)	CNS ^a^ (*n* = 214)	*Streptococcus* spp. (*n* = 93)
0	119 (65.0)	39 (11.7)	65 (68.4)	76 (35.5)	37 (39.8)
1	36 (19.7)	102 (30.6)	23 (24.2)	64 (29.9)	20 (21.5)
2	12 (6.6)	111 (33.3)	5 (5.3)	39 (18.2)	9 (9.7)
3	11 (6.0)	72 (21.6)	1 (1.1)	19 (8.9)	10 (10.8)
4	2 (1.1)	8 (2.4)	0 (0.0)	10 (4.7)	11 (11.8)
5	1 (0.5)	1 (0.3)	0 (0.0)	5 (2.3)	6 (6.5)
6	2 (1.1)	0 (0.0)	1 (1.1)	1 (0.5)	0 (0.0)
No. (%) of MDR ^b^	16 (8.7)	81 (24.3)	2 (2.1)	35 (16.4)	27 (29.0)

^a^ CNS, coagulase-negative *Staphylococcus* spp. ^b^ Multidrug resistance was defined as the acquired resistance to at least one agent in three or more antimicrobial classes.
